# Interaction of Liberibacter Solanacearum with Host Psyllid Vitellogenin and Its Association with Autophagy

**DOI:** 10.1128/spectrum.01577-22

**Published:** 2022-07-11

**Authors:** Poulami Sarkar, Murad Ghanim

**Affiliations:** a Department of Entomology, Agricultural Research Organization, Volcani Institute, Rishon LeZion, Israel; Chinese Academy of Sciences

**Keywords:** vitellogenin, autophagy, outer membrane proteins, Liberibacter, psyllid

## Abstract

*Candidatus* Liberibacter solanacearum (CLso) haplotype D, transmitted by the carrot psyllid *Bactericera trigonica*, is a major constraint for carrot production in Israel. Unveiling the molecular interactions between the psyllid vector and CLso can facilitate the development of nonchemical approaches for controlling the disease caused by CLso. Bacterial surface proteins are often known to be involved in adhesion and virulence; however, interactions of CLso with carrot psyllid proteins that have a role in the transmission process has remained unexplored. In this study, we used CLso outer membrane protein (OmpA) and flagellin as baits to screen for psyllid interacting proteins in a yeast two-hybrid system assay. We identified psyllid vitellogenin (Vg) to interact with both OmpA and flagellin of CLso. As Vg and autophagy are often tightly linked, we also studied the expression of autophagy-related genes to further elucidate this interaction. We used the juvenile hormone (JH-III) to induce the expression of Vg, thapsigargin for suppressing autophagy, and rapamycin for inducing autophagy. The results revealed that Vg negatively regulates autophagy. Induced Vg expression significantly suppressed autophagy-related gene expression and the levels of CLso significantly increased, resulting in a significant mortality of the insect. Although the specific role of Vg remains obscure, the findings presented here identify Vg as an important component in the insect immune responses against CLso and may help in understanding the initial molecular response in the vector against Liberibacter.

**IMPORTANCE** Pathogen transmission by vectors involves multiple levels of interactions, and for the transmission of liberibacter species by psyllid vectors, much of these interactions are yet to be explored. *Candidatus* Liberibacter solanacearum (CLso) haplotype D inflicts severe economic losses to the carrot industry. Understanding the specific interactions at different stages of infection is hence fundamental and could lead to the development of better management strategies to disrupt the transmission of the bacteria to new host plants. Here, we show that two liberibacter membrane proteins interact with psyllid vitellogenin and also induce autophagy. Altering vitellogenin expression directly influences autophagy and CLso abundance in the psyllid vector. Although the exact mechanism underlying this interaction remains unclear, this study highlights the importance of immune responses in the transmission of this disease agent.

## INTRODUCTION

The carrot psyllid *Bactericera trigonica* is the main insect vector that transmits *Candidatus* Liberibacter solanacearum Haplotype D (CLso) in Israel (1–3). Similar to *Candidatus* Liberibacter asiaticus (CLas), the causative agent of the devastating citrus greening disease (4), *Ca*. L. solanacearum is a phloem-limited Gram-negative bacterium, transmitted by psyllids in a persistent, propagative manner (3, 5–8). Several Liberibacter effectors like Sec-delivered effector-1 (9) and Lso-HPE-1 (10) have been identified to act as virulence factors in plants. In addition, few studies have addressed the biological and epidemiological relationships between Liberibacter species and psyllid vectors (11–17). However, little attention has been given to the molecular interactions and the functional validation of Liberibacter and insect proteins that aid in the transmission process. Recently, several reports have unraveled at the transcriptional response of whole psyllids and organs to the acquisition and retention of Liberibacter species (3, 18–21). These studies have shown that the acquisition of different Liberibacter species, by their respective psyllid vectors, induced significant immune responses (22–25). Genome and transcriptome sequencing results have further shown that psyllids do not bear a complete immune response system as has been described in model insects such as Drosophila (14, 26, 27). Psyllids lack the adaptive immunity and the immune deficiency (Imd) pathway, which generally respond to invasion by Gram-negative bacteria, thus leading for example to the ability of Liberibacter species to invade tissues in psyllids where they are able to replicate (21, 23, 28). Such findings raise the hypothesis that psyllids use alternative immune responsive mechanisms for combating with the effects of invasion by the bacterium into host cells. On the other hand, psyllids bear an innate defense mechanism against pathogens, which involves both cellular and humoral immune responses (23, 28, 29). Cellular responses include phagocytosis, and humoral responses involve secretion of several antimicrobial peptides (23, 28, 30). The first line of defense involves recognition of conserved elicitors, molecules, or essential structures often known as microbe- or pathogen-associated molecular patterns (MAMPs or PAMPs) by host pattern-recognition receptors (PRR). Bacterial outer membrane proteins (Omp), flagellins, and pili appendages are some of the known bacterial virulence factors involved in pathogenesis that elicit immune responses in the host (31–33). OmpA is a major unique integral transmembrane protein with amphipathic β-barrels which is often involved in cell adhesion and virulence (34–37). OmpA also has a direct role in virulence upon infection in the host cells for several human-pathogenic bacteria (36, 38) such as Escherichia coli (39, 40), Salmonella enterica (41), Leptospira interrogans (42, 43), and Neisseria gonorrhoeae (44). The fat body in insects is one of the major immune-responsive organs, where host PRRs against bacterial virulence factors are produced and then directly released into the hemolymph (45). Additionally, hemocytes act as macrophages that have phagocytic activity but also require the presence of PRRs for presenting the pathogen to these macrophages.

One of the major known PRRs is apolipoprotein or vitellogenin (Vg) which belongs to the large lipid transfer protein (LLTP) superfamily having opsonin activity (46–48). LTTPs consist of a large phosphoglycolipoprotein and a major egg yolk protein precursor (YPP) in insects. They are large molecules (200 kDa) synthesized in the fat bodies and midguts, transported through the hemolymph and sequestered by ovaries with the help of vitellogenin receptors (VgR) via receptor-mediated endocytosis, and are subsequently cleaved to generate the nutrient yolk protein vitellin required for the developing oocytes (49–51). Although Vg was initially considered a female-specific protein, males and sexually immature animals have also been shown to express Vg indicating several roles beyond the nourishment of developing oocytes (50, 52, 53). It provides host innate immunity with multifaceted functions during several extraneous factors, including chemical exposure, nutritional stress, and infection (47, 54). Insect Vg often acts as a pattern recognition molecule to recognize pathogens, enhances macrophage phagocytosis and autophagy, neutralizes viruses by creating cross-links between virions, and often kills bacteria by interacting with the lipopolysaccharides and lipoteichoic acid present in bacterial cell walls (49, 51, 53, 55, 56). For instance, silkworm apolipoproteins inhibits Staphylococcus aureus by binding to cell surface lipoteichoic acids (57, 58). Mosquito Vg has been reported to have antiparasitic response against plasmodium (59). Bacterial membrane proteins, flagella, and pili often serve as PAMPs and immune elicitors that interact with Vg and act as PRRs which induce autophagy and transgenerational immune priming (36, 60–65).

In this study, we show that OmpA and flagellin of CLso interact with Vg of *B. trigonica* and induce autophagy in the psyllid cells. While both Vg and autophagy are important in the immune response against CLso, each seems to negatively regulate the other, and both are important for regulating CLso titers, in addition to oocyte development, oviposition, and egg viability. The described immune responses in this study are crucial for CLso persistence in the insect and seem to be part of a larger mechanism regulated by both the insect and CLso for maintaining the balance between the vector and the pathogen.

## RESULTS

### Liberibacter surface proteins interact with the von Willebrand factor type D (VWD) domain of host vitellogenin.

**FIG 1:**
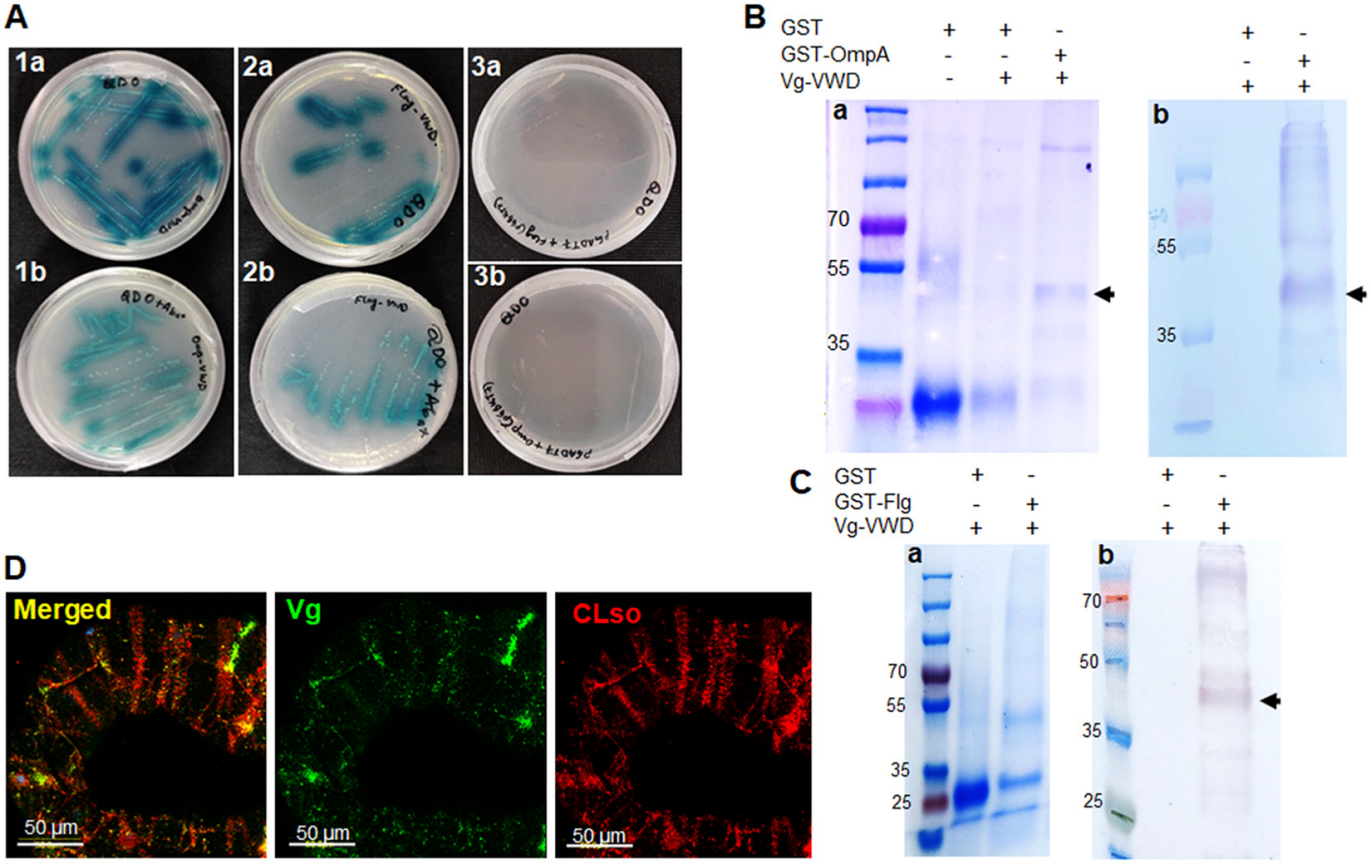
Interaction between Liberibacter membrane proteins and host vitellogenin. (A) Yeast two-hybrid assay showing strong interaction between Vg-VWD domain and bacterial OmpA (1a, 1b) and Flagellin (2a, 2b) in QDO+Xgal (a) and QDO+Xgal+Aba plates (b). Subsets 3a and 3b show negative control showing no interaction between empty pGADT7 vectors with bacterial proteins. (B) and (C) Detection of Vg-VWD with N-terminal His-tag after pulldown assay using GST-tagged OmpA (B) and Flg (C) as baits by SDS-PAGE (a) and Western blot (b) using anti-His antibody. (D) Immunostaining of *Ca.* L. solanacearum+ midguts with anti-Vg antibody (green) and anti-*Ca.* L. solanacearum antibody (red) showing spatial colocalization of the two (yellow) under confocal microscopy.

As OmpA contains a surface antigen domain, while Flg acts as a virulence factor (31), Liberibacter OmpA and Flg were used to screen for interacting proteins in the psyllid vector. A cDNA expression library was prepared using whole psyllids and was mated with the full-length CDS of OmpA/Flg expressed as a fusion protein with GAL4-DNA binding domain in Y2HGold that binds to promoters of four reporter genes (AUR1C, HIS3, ADE2, and MEL1). Around 54 isolated colonies were obtained in QDO plates for OmpA and 62 for Flg which were restreaked on QDO/Xgal and QDO/X-gal/Aba plates for confirmation of β-galactosidase activity. Four out of all the colonies for OmpA and three for Flg were identified as parts of VWD domain of Vg after DNA sequencing (Fig. S3). To verify the Y2H interaction, VWD domain of Vg was amplified separately from psyllid DNA, cloned into pGAD-T7 vector, and screened against OmpA/Flg once again which also showed strong β-galactosidase activity (Fig. 1A).

Interaction between Vg and OmpA/Flg was further confirmed using a pulldown assay using OmpA as a bait. A band of approximately 48 kDa was observed in SDS-PAGE as well as Western blots using a monoclonal Anti-polyHistidine antibody produced in mouse (Sigma-Aldrich, Israel) when OmpA and Vg were included in the assay (Fig. 1B) or when Flg and Vg were used (Fig. 1C). No band was detected when GST control was used as bait, indicating a specific interaction between OmpA/Flg and Vg-VWD. To further confirm the interaction, spatial localization of Vg was confirmed in dissected midguts from *Ca.* L. solanacearum infected psyllids using immuno-localization with specific antibodies for Vg and *Ca.* L. solanacearum. The signal observed in the midgut indicated a partial overlap in the fluorescent signals of Vg and *Ca.* L. solanacearum, which indicated a physical proximity in midgut cells (Fig. 1D). Vg localization and expression profile in *Ca.* L. solanacearum-infected and *Ca.* L. solanacearum-free males and females are shown in Fig. S4.

### *In silico* analyses.

**FIG 2:**
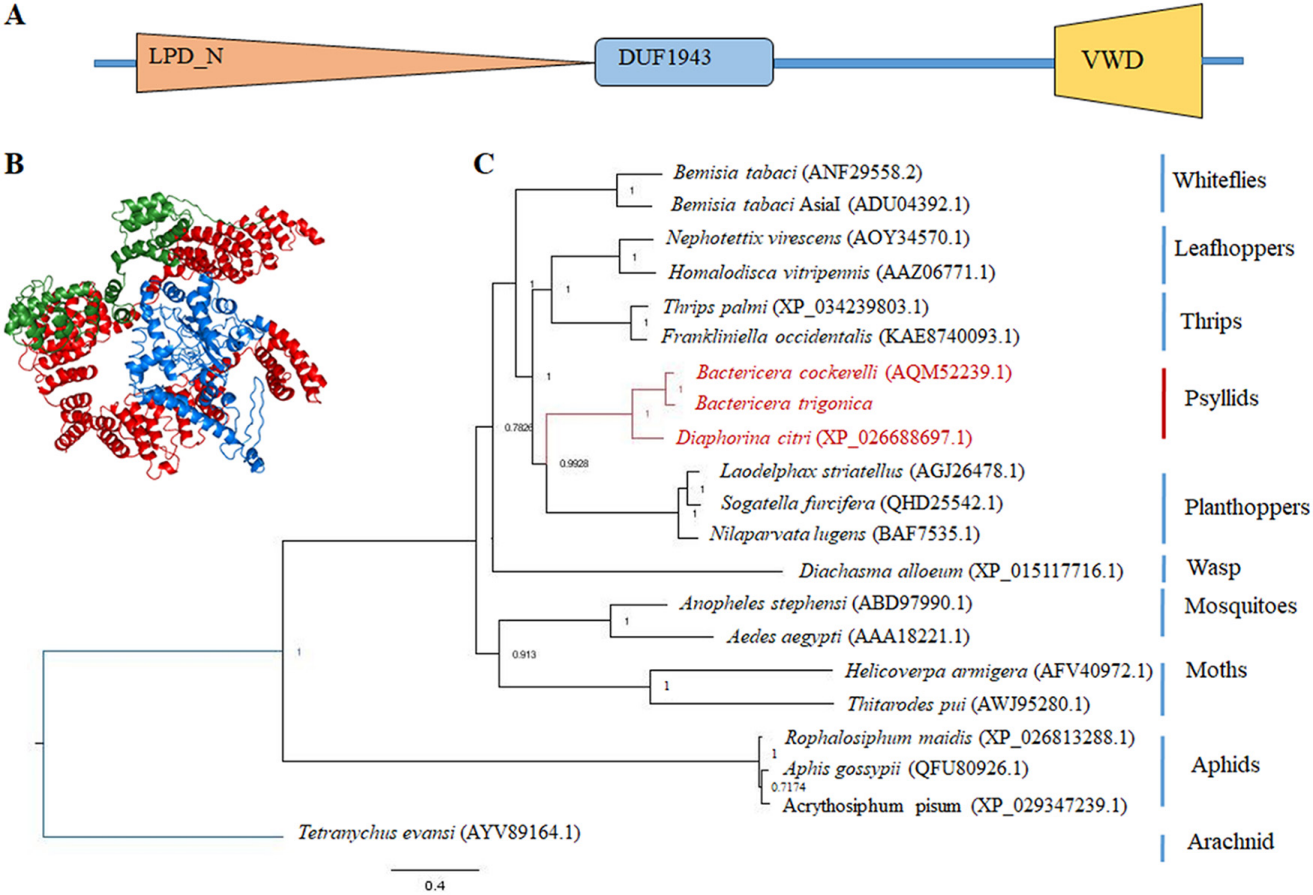
Structure and domain architecture of vitellogenin. (A) Vg contains three conserved domains; Lipoprotein LPD_N-terminal domain, DUF1943, and VWD domain. (B) Three-dimensional structure of psyllid Vg, modeled by iTasser with highest C-score of 0.32 shows the two major domains: LPD_N (red) and VWD (blue). (C) Phylogeny of amino acid sequences of all known psyllid Vg proteins showing clustering within Hemipteran clade with *T. evansi* used as an outgroup.

Only one Vg homolog was identified from the psyllid transcriptome (3). The coding sequence was validated by cloning and sequencing. Structural analysis of Vg revealed three major domains, which include Lipoprotein_N-terminal domain (LPD_N), a 1943 domain (DUF1943) of unknown function, and C-terminal von Willebrand factor type D domain (VWD) that are usually found in conventional Vgs/LLTPs (Fig. 2A and B). Phylogenetic analysis showed that the carrot psyllid Vg clustered in the same clade as two other psyllids with 90.7% identity with the potato psyllid *Bactericera cockerelli*. It also clustered in a separate clade formed by other hemipterans in the group (Fig. 2C).

Amino acid sequences of all known Liberibacter OmpA and Flg were aligned for sequence identity. Despite an overall high level of identity, conservation of sequences were found to be scattered for both OmpA and Flg. Domain analysis for OmpA revealed four polypeptide transport-associated domain (POTRA) and a bacterial surface antigen. Homology modeling also revealed a three-dimensional structure for OmpA with all the major domains (Fig. S5). Similar analyses for flagellin revealed a signaling domain and a polymerization domain and show minor heterogeneity when aligned with conserved sequences (Fig. S6).

### *Ca.* L. solanacearum induces vitellogenin and autophagy-related genes in psyllids.

**FIG 3:**
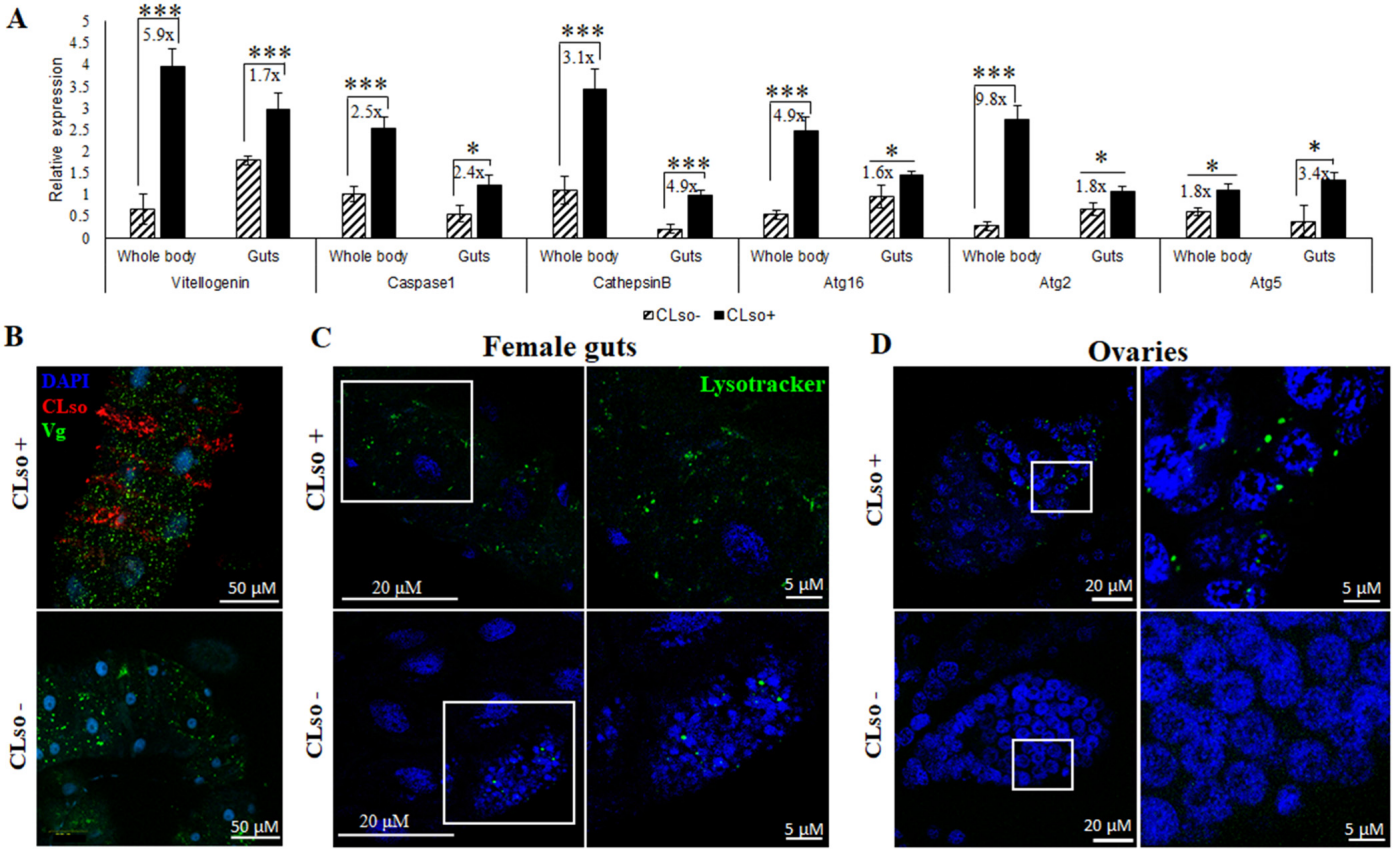
Expression profiles of vitellogenin (Vg) and the autophagy-related (Atg) genes CaspaseI, Cathepsin B, Atg16, Atg2, and Atg5 in *Ca.* L. solanacearum uninfected and infected psyllids. (A) Relative expression using real-time PCR showing upregulated gene expression of Vg and Atg-genes in *Ca.* L. solanacearum+ whole bodies and midguts compared to *Ca.* L. solanacearum-psyllids (*, *P* < 0.05; ***, *P* < 0.001). (B) Higher expression of Vg (green) in *Ca.* L. solanacearum-infected midguts compared with *Ca.* L. solanacearum-free as seen using immunostaining. (C, D) Staining of acidic compartments (lysosomes and autolysosomes) using LysoTracker Green showing higher lysosomal activity in *Ca.* L. solanacearum+ midguts (C) and in ovaries (D) compared with *Ca.* L. solanacearum-psyllids.

Differential expression profiles of Vg and autophagy related genes were studied in *Ca.* L. solanacearum-free and *Ca.* L. solanacearum-infected psyllids. Immunostaining revealed higher expression of Vg in the *Ca.* L. solanacearum-infected midguts and its expression was upregulated in *Ca.* L. solanacearum-infected psyllids compared with control *Ca*. L. solanacearum-free psyllids by 5.9-fold changes in whole body samples (in both males and females) and by 1.75-fold changes in the midguts (Fig. 3A and B).

Cathepsin-B and Caspase-I, known immunity genes involved in lysosomal functions against several pathogen invasions, were found to be upregulated in *Ca.* L. solanacearum-infected psyllid whole body and midguts. Additionally, the expression of autophagy genes Atg16, Atg2, and Atg5 involved in autophagosome formation was also upregulated in both midguts and whole psyllids (Fig. 3A). Higher lysosomal activity in *Ca.* L. solanacearum-infected midguts (Fig. 3C) and ovaries (Fig. 3D) compared with *Ca.* L. solanacearum-free psyllids was observed when guts and ovaries were stained with Lysotracker, which specifically binds to acidic organelles, indicating higher formation of autolysosomes. The intensity of the Lysotracker signal in *Ca.* L. solanacearum-infected midguts were 3.12 ± 1.2 times more than *Ca.* L. solanacearum-free as validated using integrated intensity in ImageJ software.

### Inducing vitellogenin impairs autophagy and vice versa.

**FIG 4:**
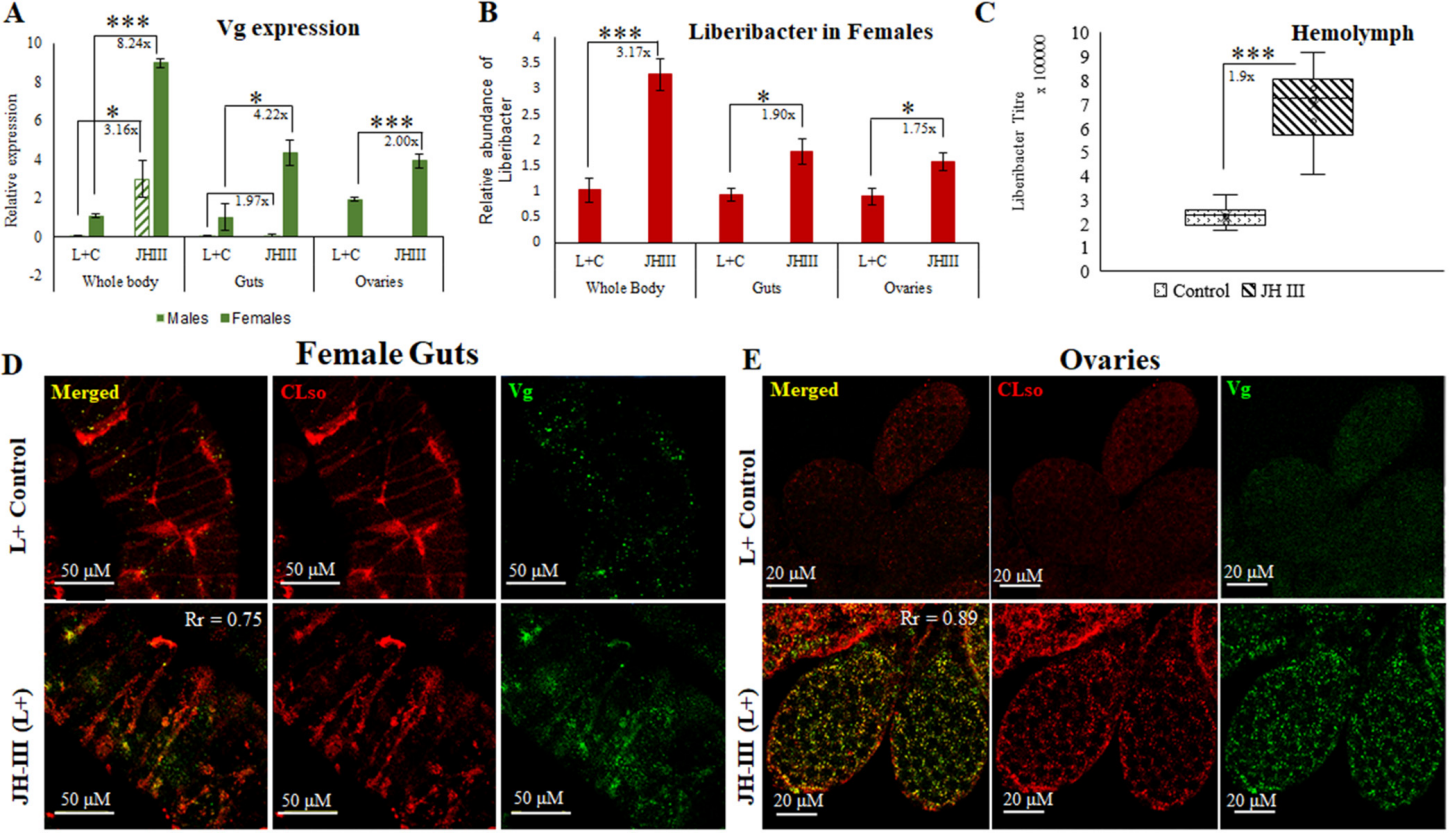
Effect of JH-III hormone on Vg and *Ca.* L. solanacearum. (A) Relative expression of Vg in male and female whole body, midguts, and in ovaries showing induced expression of Vg throughout with females showing much higher expression than in males, upon JHIII application than in control (L+C) (*, *P* < 0.05; ***, *P* < 0.001). (B) Relative titer of *Ca.* L. solanacearum (Omp) in female whole bodies, midguts, and in ovaries after JH-III application (*P* ≤ 0.05). (C) Elevated *Ca.* L. solanacearum titer in the hemolymph of JH-III treated psyllids (*, *P* < 0.05; ***, *P* < 0.001). The fold change for each gene is mentioned beside the bars. (D, E) Immunostaining of Vg and *Ca.* L. solanacearum showing induction of Vg (green) expression and increase in *Ca.* L. solanacearum titer (red) upon JH-III application along with their colocalization. The colocalization was validated using ImageJ with Pearson’s correlation coefficient (R value) of 0.75 and 0.89 for guts and ovaries, respectively.

**FIG 5:**
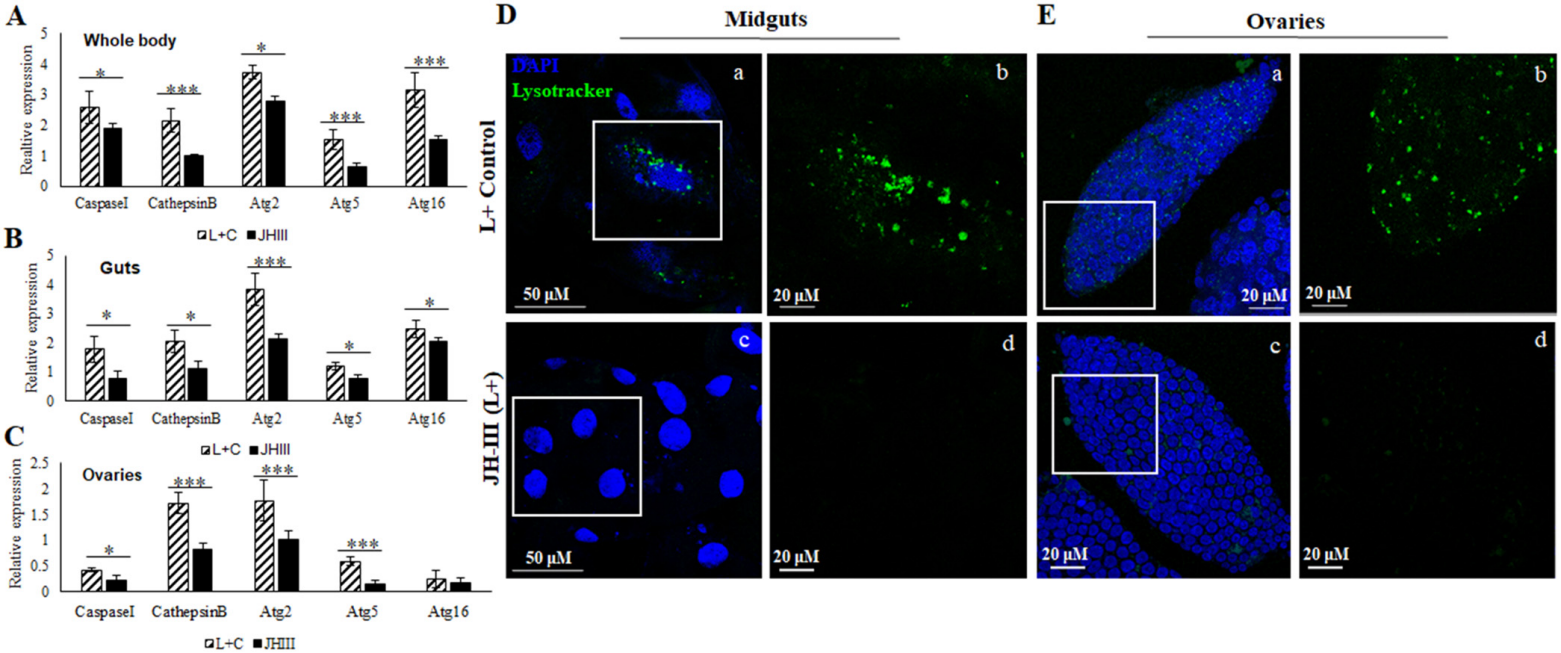
Effect of JH-III on autophagy. Relative expression of lysosomal and autophagy genes in whole bodies (A), midguts (B), and ovaries (C) showing downregulation of all the known genes upon JH-III application (*P* ≤ 0.05). (D, E) Representative images showing reduction of autophagy and lysosomes in the JH-III applied psyllids. Staining of the midguts (D) and ovaries (E) with DAPI (blue) and lysosomes (green) with b and d showing magnified images of the insets in a and c, respectively. *, *P* < 0.05; ***, *P* < 0.01.

Vg expression was measured following psyllid treatment with the JH-III hormone, which is the main regulator of Vg production during oogenensis. After 16 h of exposure to JH-III, significant elevation of Vg expression was observed in whole bodies and midguts of both male and female with significantly higher induction in females (Fig. 4A). Female psyllids in which Vg was induced also had increased number of fat bodies as seen during dissection (data not shown). Induction of Vg also induced Liberibacter titer in the midguts as well as in the hemolymph as measured by qPCR and immunostaining (Fig. 4B to D), where elevated autolysosomal activities were also observed (Fig. 3). Vg and *Ca.* L. solanacearum were seen to mostly colocalize in midguts and ovaries as validated by Pearson’s correlation coefficient (*R* >0.75). Induction of Vg expression, however, caused a significant downregulation of the autophagy-related genes in whole females (Fig. 5A) midguts (Fig. 5B) and ovaries (Fig. 5C). The presence of autolysosomes was almost negligible in the JH-III treated psyllid midguts (Fig. 5D) and ovaries (Fig. 5E) compared with the control treatments.

**FIG 6:**
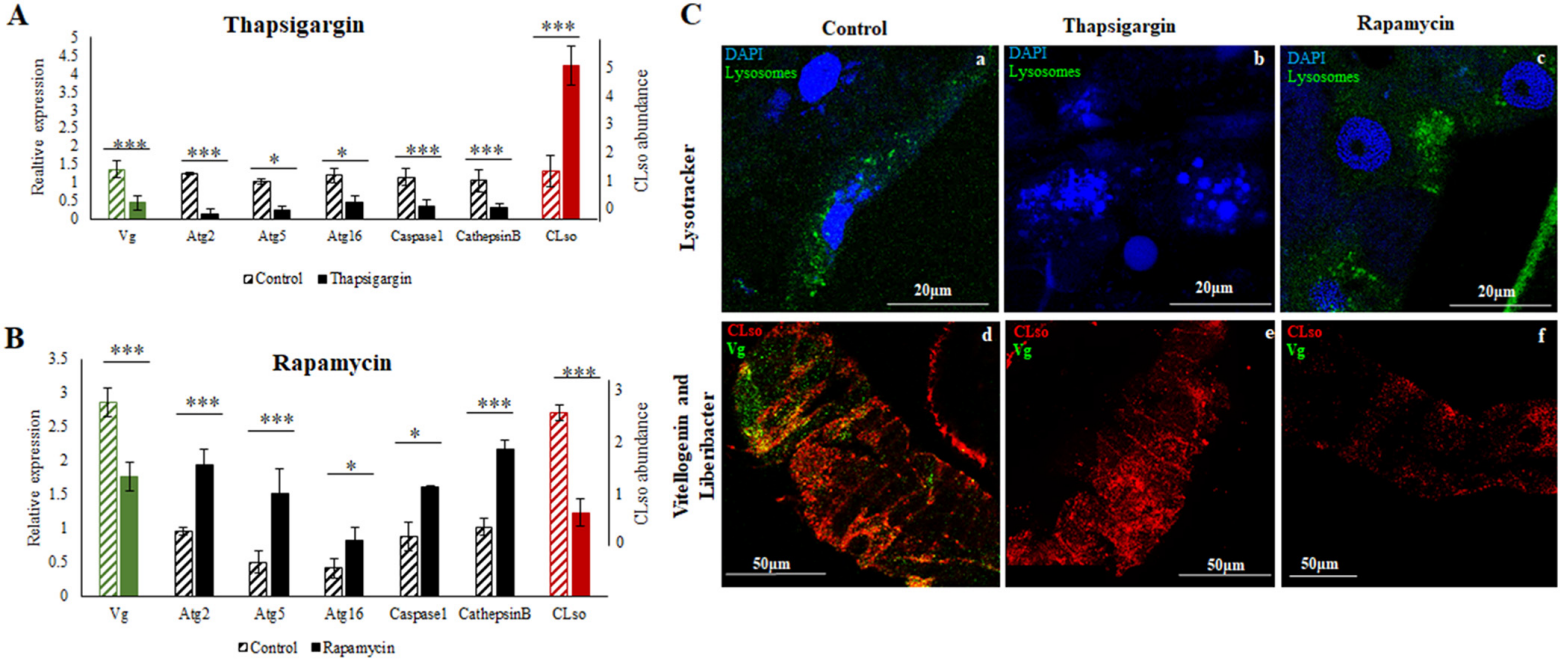
Effect of Thapsigargin and Rapamycin on Vg, *Ca.* L. solanacearum, and autophagy. (A) Relative gene expression of Vg, autophagy genes and *Ca.* L. solanacearum titer in the psyllid midguts upon Thapsigargin application (*P* < 0.05). (B) Relative gene expression of Vg, autophagy genes, and *Ca.* L. solanacearum titer in the psyllid midguts upon Rapamycin application (*P* < 0.05). (C) Staining of lysosomes (green) and nuclei (blue) showing disintegrated nuclei and absence of autophagy upon Thapsigargin application (b), and increase in lysosomal activity upon Rapamycin application (c) compared with the control midguts (a). Lower panel showing decrease in vitellogenin and increase in *Ca.* L. solanacearum titer upon Thapsigargin application (e) and lower *Ca.* L. solanacearum abundance upon Rapamycin application (f) compared with control midguts (a). *, *P* < 0.05; ***, *P* < 0.01.

Interestingly, application of thapsigargin, that specifically inhibits autophagy, reduced the expression of Vg along with all other autophagy genes, while causing an increase in *Ca.* L. solanacearum levels, as seen in qRT-PCR (Fig. 6A) and immunostaining (Fig. 6C). On the other hand, using the specific autophagy inducer rapamycin significantly induced autophagy and autophagy related genes and reduced the expression of Vg and Liberibacter titers in the psyllid midguts (Fig. 6B and C).

### Induction of Vg impairs egg development, oviposition, and viability.

**FIG 7:**
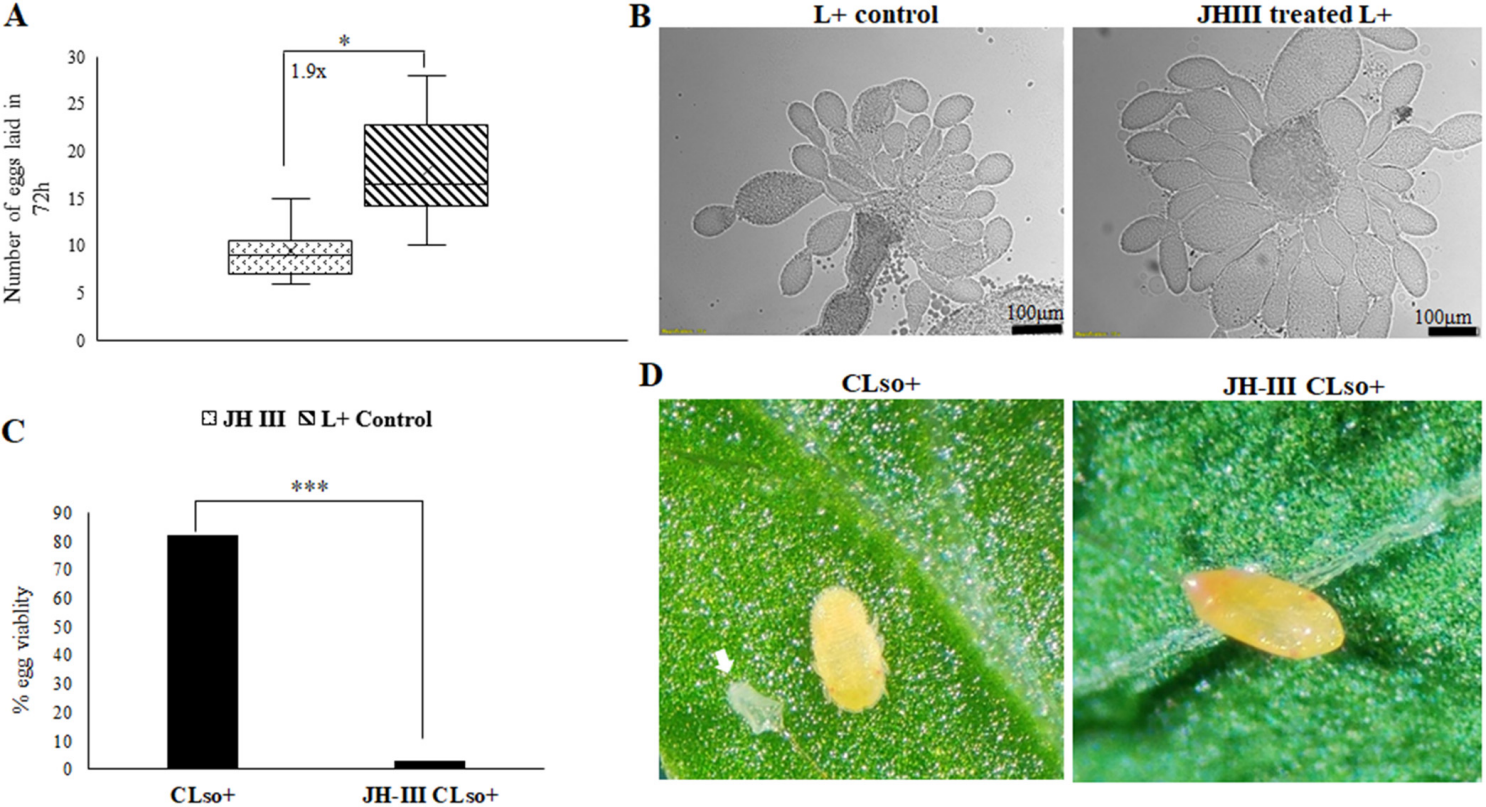
Effect of JH-III on egg development and viability. (A) JH-III application reduces oviposition (*, *P* < 0.001). (B) Representative images showing increased number of ovarioles developing in ovaries dissected from females that were exposed to JH-III. (C) Number of hatched eggs (fertility) is significantly reduced upon JH-III treatment compared with control psyllid eggs. (D) Representative image showing a nymph hatching from *Ca.* L. solanacearum+ control egg and a dehydrated egg laid by JH-III treated females. White arrow shows the egg shell from which the nymph hatched.

Because Vg has an important role in oogenesis and egg development, we investigated its induction following JH-III treatment on mortality, oviposition, and fertility, compared with induction as a result of the presence of *Ca.* L. solanacearum. No significant mortality was observed in the JH-III exposed female psyllids compared to the controls. However, a significant reduction in the number of eggs laid by Vg induced female psyllids was obtained (Fig. 7A). JH-III application further induced oocyte development in female psyllids post-48-h treatment with higher number of mature oocytes that was observed compared with that of control females (Fig. 7B). Moreover, only 3% of the eggs laid by JH-III exposed females hatched compared with 83% viability in *Ca.* L. solanacearum-infected control eggs (Fig. 7C), with observed malformations and developmental defects in the laid eggs following JH-III treatment (Fig. 7D).

### Absence of transovarial transmission.

Because JH-III significantly induced *Ca.* L. solanacearum titers and ovary development in the psyllids, we tested whether these effects may cause the bacterium to be transferred to the developing oocytes by transovarial transmission. One out of 25 hatched nymphs that developed from eggs laid by *Ca.* L. solanacearum-infected females that were reared on *Ca.* L. solanacearum-free leaves tested positive for *Ca.* L. solanacearum, indicating very low or negligible transovarial transmission. Newly hatched nymphs that developed from eggs laid by *Ca.* L. solanacearum+ females that were reared on *Ca.* L. solanacearum leaves, were completely viable and efficiently acquired *Ca.* L. solanacearum when fed on *Ca.* L. solanacearum-infected leaf flush. Interestingly, ovaries dissected from *Ca.* L. solanacearum-infected females treated with JH-III all tested positive for *Ca.* L. solanacearum (Fig. S7); however, the eggs laid were 100% unviable (Fig. 7C and D), suggesting that JH-III treatment indeed accelerates *Ca.* L. solanacearum penetrance into ovaries; however, this treatment is fatal for the eggs as it impairs autophagy.

## DISCUSSION

Bacterial membrane proteins have virulence properties and often act as pattern recognition molecules which induce host immune response (32, 61). In this study, we used Liberibacter outer membrane protein (OmpA) and flagellin (Flg) as baits to screen for specific insect host proteins that interact with OmpA and Flg. Because both of these bacterial proteins bear an important role in adhesion and virulence, we expected that both proteins will interact with psyllid proteins either for adhesion during the transmission process or for bypassing the host immunity. Yeast two-hybrid assays using OmpA/Flg as baits revealed their interaction with the host Vg-VWD domain when screened against psyllid cDNA library (Fig. 1A). Because OmpA has a transmembrane domain, we used OmpA as a prey and used Vg-VWD as a bait to revalidate the interaction. Pulldown assays and spatial coimmunolocalization using confocal microscopy also indicated specific interactions between both bacterial proteins and Vg (Fig. 1B and C). The reason behind this spatial localization of Vg is that Vg is a secretory protein which is produced in cells and is often translocated to the midgut lumen and hemolymph. However, presence of Vg in the midgut epithelial cells help in pathogen movement across the midgut cells and in some cases opsonization (66–68). Further studies are needed to confirm the interaction in the lumen or in the hemolymph which can help us with a better rationale behind CLso-Vg interaction. In the Asian citrus psyllid *Diaphorina citri*, the expression of Vg is reported to be induced in response to CLas infection (69), similar to its induction in the potato psyllid upon CLso infection (18). Vg has also been reported to act as a pattern recognition molecule against pathogens and it has been shown to mediate their degradation by hemocytes through phagocytosis (59, 60, 70, 71). Both bacterial proteins also carry immune elicitors and are able to induce immunity in subsequent generation through the process known as transgenerational immune priming (TGIP) (30, 62, 72, 73). In this study, only one Vg homolog was identified from the carrot psyllid transcriptome (3) and the full-length coding sequence was assembled and cloned. It has two major domains: apolipoprotein domain (LPD_N), which helps in lipid transport; and VWD domain, a multifunctional domain involved in maintaining homeostasis (74, 75). Based on phylogenetic analysis, Vg clustered with the other two psyllid Vgs that clustered within the hemipteran clade (Fig. 2). The surface antigen region of OmpA (Fig. S5) and hypervariable region of flagellin (Fig. S6) are reported to possess adhesion-like properties and they often act as microbe-associated molecular pattern (MAMP) inducing virulence (35–37, 39, 76).

The known interaction between Vg and bacterial membrane proteins was a trigger to investigate the role of Vg in Liberibacter pathogenesis and host immunity response. The function of vitellogenin is often known to accompany programmed autophagy during development and under stress conditions, and a tight link between the two has been reported in various studies (56, 77, 78). In this study, we investigated the gene expression profiles of autophagy-related genes (available from the transcriptome) and the presence of autolysosomes in *Ca.* L. solanacearum+ psyllid midguts and ovaries as a result of Vg induction. The results showed a significant upregulation of Vg along with the autophagy-related genes (Atg2, Atg5, and Atg16) in *Ca.* L. solanacearum+ whole body as well as in midguts of carrot psyllids (Fig. 3A and B). The midgut, being the first barrier for *Ca.* L. solanacearum, is the major organ important for transmission and the first cellular organ to meet the pathogen where it invades intracellularly and activates immune responses. Thus, it is the most suitable organ for studying the interaction between the bacteria and psyllid proteins. Although the immune system is activated in the whole body, the molecular interactions in the midgut are expected to be more intense and possibly prime other responses in other parts of the body. In the midgut, the pathogen activates machineries for adhesion, cell invasion, and crossing the basal lamina to reach the hemolymph while avoiding the psyllid defenses (3, 16). Cathepsin B and caspase I involved in lysosomal activity were also upregulated in *Ca.* L. solanacearum+ psyllids (Fig. 3B). The presence of lysosomal bodies and autolysosomes were evidently higher in *Ca.* L. solanacearum+ psyllid midguts and ovaries (Fig. 3C and D). A higher number of lysosomes indicates higher lysosomal activity, and higher autophagy as autophagosomes delivers cytoplasmic materials or cellular debri to the lysosomes for degradation. These results explain the joint and orchestrated function of both Vg and autophagy-related genes upon Liberibacter infection for maintaining homeostasis, and the crucial role of these functions for maintaining the cell viability. However, when Vg expression was induced with the application of JH-III hormone (Fig. 4), there was a drastic reduction in autophagy and lysosomal activity (Fig. 5), and the expression of autophagy-related genes and lysosomal proteases were significantly downregulated (Fig. 4 and 6). Moreover, Vg induction drastically reduced oviposition and egg viability (Fig. 7). JH-III is a well-known regulator of vitellogenesis (79, 80). It is known that overexpression of Vg induces ageing and impairs the induction of autophagy and lysosomal genes required to maintain longevity (78) and autophagy is induced during the synthesis phase of Vg in the fat body to maintain developmental switches, regulate immunity, and recycle cellular components during development (77, 81, 82). On the other hand, upon autophagy arrest by thapsigargin, Vg expression was reduced along with the autophagy-related gene expression in the psyllids (Fig. 6A and C). This was surprising to us although the result is in congruence with previous reports where it has been shown that cellular calcium plays a basal regulatory role in Vg production independent of mTOR pathway which is the major regulator for autophagy as well as for Vg expression. Thapsigargin acts as calcium mobilizing agent while blocking autophagy, while inducing both ER stress and apoptosis (83–86). This induction of apoptosis is also known to activate Perk-eIF2 pathway and Ire-1 dependent decay (RIDD) of mRNA, which results in reduced synthesis and degradation of Vg mRNA, respectively (83, 87). Additionally, Liberibacter titers increased significantly, and its signal was seen to be diffused in the psyllid midguts treated with thapsigargin. This experiment helped us solely to understand the effects of reduced autophagy on CLso abundance. Further studies can be done to silence specific autophagy-related genes to understand how Vg and autophagy process are connected. Interestingly, inducing autophagy and blocking the mTOR pathway by applying rapamycin, reduced Vg expression as well as Liberibacter in the psyllid midguts. The reduction in Liberibacter titer is believed to be a result of increased autophagy. This result is in correspondence with new findings in ticks where vitellogenesis is delayed by the application of rapamycin and is regulated by autophagic mTOR pathway (88). Overall, we know that Vg induction is mostly regulated by mTOR than by a calcium-regulated pathway. Disrupting these two pathways independently have similar effects on Vg, although, the negative effects of autophagy on CLso remains constant. As programmed autophagy is crucial for proper cell development, it will be interesting to study how Vg and autophagy regulate each other during egg maturation in psyllids. This indicates that both vitellogenesis and autophagy are important for cell survival and are integral parts of developmental process, which help in maintaining cellular homeostasis. Any imbalance between the two may disrupt the homeostasis and may lead to cell death (77, 78, 89, 90).

The results of this study also show elevated titers of Liberibacter in the JH-III-treated midguts as well as in the hemolymph in the absence of autophagy (Fig. 4 and 6). Higher abundance of Liberibacter titer in the midguts and hemolymph suggests a role of Vg in presenting Liberibacter to the cells inducing autophagy, whose absence results in higher titers of the pathogen in the system. There might also be a role for Vg in transgenerational immune priming in *Ca.* L. solanacearum-infected psyllids and a possibility of transovarial transmission in the absence of autophagy. We could not detect *Ca.* L. solanacearum in viable ovaries and laid eggs, which indicates the absence of transovarial transmission. Nymphs developing from eggs laid by *Ca.* L. solanacearum-infected females that hatched and fed on *Ca.* L. solanacearum leaves were also negative for *Ca.* L. solanacearum. This implies that nymphs acquire *Ca.* L. solanacearum by feeding only on infected leaves with *Ca.* L. solanacearum, and not by transovarial transmission. Surprisingly, ovaries dissected from *Ca.* L. solanacearum+ females which were treated with JH-III tested positive for Liberibacter in the absence of autophagy. However, induction of Vg reduced egg viability although vitellogenic development in the oocytes and the number of ovarioles was greater compared with the control *Ca.* L. solanacearum+ psyllid ovaries (Fig. 7). This possibly happened due to the lack of autophagy, which disrupted proper cellular development. These results suggest that the ovaries tested positive for *Ca.* L. solanacearum because of Vg induction or reduction in autophagy. These results suggest a role for Vg in the defense mechanism and might be involved in TGIP, although an exact mechanism remains unknown. Although, the correlation between Vg and autophagy was tested in females with respect to oviposition and egg viability, it will be interesting to see if this interrelation and variability in expression is similar in the males. It will also be exciting to compare the immune response and liberibacter transmission competence between the male and the female psyllids following liberibacter acquisition.

In summary, the results presented in this study reveal that both vitellogenin and autophagy are essential in regulating *Ca.* L. solanacearum levels and possibly its persistence, transmission, and generated stress responses in the psyllid cells. Although the role of autophagy in CLso abundance seems to be inversely proportional, the exact role of Vg remains unclear. We believe one of the reasons behind CLso-Vg interaction is to regulate autophagy for easy pathogen persistence which also helps in maintaining a homeostasis between vitellogenesis and autophagy for host survival. It will be interesting to study how Vg interacts with autophagosomes or autophagy-related targets upon binding with CLso. This will further explain the relationship between vitellogenesis and autophagy process in psyllids upon CLso infection. Future studies are imperative to investigate whether Liberibacter interacts with vitellogenin to manipulate the host immune response for its survival, or it is a host defense mechanism against Liberibacter to reduce cellular stress and maintain homeostasis.

## MATERIALS AND METHODS

### Maintenance of psyllid and Liberibacter.

*Ca*. L. solanacearum-infected and *Ca*. L. solanacearum-free psyllids were maintained on 2 months old Parsley (*Petroselinum crispum*) in separate rooms, under 14-h photoperiodic light at 25 ± 2°C. The plants as well the psyllid population were tested for *Ca*. L. solanacearum routinely.

### Plasmid vectors.

For protein expression studies, we used pRSET-A and pFN2A Flexi vectors (Thermo Scientific) with competent BL21(DE3) and DH5α cells (NEB, USA). pGAD-T7 Rec (Clontech) was used for the cDNA library preparation, pGADT7-AD (Clontech) as prey vector for one to one assays, and were then transformed in Y2H Gold yeast cells. pGBKT7 (Clontech) was used as a bait vector (DNA-BD) and was transformed into Y187 yeast cells.

### Yeast two-hybrid bait constructs.

Sequences of Liberibacter Outer membrane protein (OmpA) and Flagellin (Flg) were derived from the full genome sequence of Candidatus *Liberibacter solanacearum* (Haplotype D) with accession PKRU02000006.1 (91). Full-length coding sequence of OmpA and Flg were amplified from Liberibacter infected (CLso+) psyllids using Q5 DNA polymerase (NEB, USA), cloned into the bait vector-pGBKT7 (EcoRI/BamHI) using In-Fusion HD cloning kit (TaKaRa) and screened for positive recombinants in DH5α cells. Recombinant OmpA-pGBKT7 and Flg-pGBKT7 were finally transformed into yeast two-hybrid Gold yeast strain separately using Yeastmaker yeast transformation system (TaKaRa, Clontech).

### Psyllid library construction.

Total RNA was extracted from around 100 CLso+ psyllids using TRIzol (Sigma) and purified using RNAeasy kit (Qiagen). First-strand cDNA was synthesized with 3.6 μg of total RNA using Make your own “mate & plate” library system (TaKaRa, Clontech) according to the manufacturer’s instruction. The first-stranded cDNA was next amplified to produce double-stranded cDNA in 20 amplification cycles by long-distance PCR using the Advantage 2 polymerase mix (TaKaRa, Clontech) following the manufacturer’s instructions. The double-stranded cDNA was purified using Chroma Spin+TE-400 to eliminate any products below 200 bp. The purified ds cDNA was finally cotransformed with pGAD-T7 Rec into competent Y187 using Yeastmaker yeast transformation system (TaKaRa, Clontech) and plated on SD-Leucine agar media. The plates were incubated at 30°C for 3 to 5 days. Around 2.6 million independent cDNA clones were obtained and the colonies were pooled using YPDA freezing media and stored in aliquots in −80°C.

### Y2H assays.

The two baits, OmpA-pGBKT7 and Flg-pGBKT7, were tested for self-activation and were further screened against the psyllid library individually, following Matchmaker Gold yeast two-hybrid user manual (TaKaRa, Clonetech). A culture of the bait (Y2HGold) was allowed to mate with psyllid library for 24 h, and after mating, the cells were plated on QDO (SD-ATLH) media and incubated at 30°C for 8 to 10 days. Developed colonies were restreaked onto QDO/X-gal^+^ plates to screen for the development of blue color for the β–galactosidase activity, and finally the blue colonies were further streaked onto QDO/Xgal/Aureobasidin (40 μg/mL). Plasmids were isolated from the colonies as previously described (92) and sequenced for identity.

### RNA extraction, qRT-PCR analysis, and Liberibacter abundance.

Single psyllids/guts/ovaries were used for RNA and DNA extraction, both from the same sample using CTAB (93) and as previously described (94). Males (1 week old) were only used for testing the expression of Vg. Females were used for all other experiments. The guts and ovaries were washed three times with PBS to remove any contaminants from the hemolymph before proceeding with RNA/DNA extraction. Final eluted nucleic acid was divided into two aliquots, one for RNA and one for DNA. DNA contaminations from the total RNA were removed with DNase I (Thermo) and used for cDNA synthesis using Verso cDNA synthesis kit (Thermo) following manufacturer’s instructions. RNA was removed from the DNA sample using RNase I (Thermo) and was used to measure relative Liberibacter titer using qPCR. Real-time analyses were carried out using 2x Absolute Blue SYBR mix (Thermo) and 1 μL of diluted cDNA in a final volume of 20 μL. Threshold Ct values were calculated in StepOne real Time PCR system (Applied Biosystems) and normalized using the housekeeping genes (elongation factor). PCR efficiencies of all new primers were tested using a standard curve, and differential gene expression were analyzed using 2^−ΔΔCT^ quantitation methods (95). The primers used in this study are listed in Table 1. Statistical analyses of all qRT-PCR data were conducted by one-way ANOVA with Tukey’s *post hoc* test (*P* < 0.05). The accumulation of *Ca.* L. solanacearum in the hemolymph was also quantified with the method previously described (94) using a Nanoliter 2010 injector (World Precision Instruments, Sarasota, FL, USA). The hemolymph was diluted in 10 μL of water and was used directly for qPCR analysis with OmpA-specific primers. Statistical analysis was done using Student's *t* test (*P* < 0.05).

**TABLE 1:** Primers used for PCR detection, qRT-PCR

Primer name	Sequence 5′ > 3′	Target	Product size (bp)	Reference
Primers used for qPCR				
Ef1α_Fq	CCACCACCAACACATCTAC	*B. trigonica* elongation factor 1α	119	109
Ef1α_Rq	ACTTCTTCTCCTCCTCTCATCT			
qVWD_F	GAGAACCACAATTCCGTCAAT	*B. trigonica* Vg	197	This study (GenBank MW316414)
qVWD_R	TTTGGGCGGAAGATCCATC			
qOmp_F	CCATATCCAAATTTCAAAGAACC	*Ca.* L. solanacearum ompA	152	110
qOmp_R	ATGCCACGTGAAGGTTTGAT			
Actin_F	AGATGACCCAGATCATGTTTGA	*B. trigonica* Actin	140	110
Actin_R	AGGGCGTAACCTTCATAGATG			
CathB_Fq	CAAGTCTGGTGTGTACAAGCA	*B. trigonica* CathepsinB	125	This study (GenBank OK188783)
CathB_Rq	TGTTCCACGAATTGGCGATC			
CaspaseI_Fq	GTCTGGGAGAACGCTACC	*B. trigonica* CaspaseI	165	This study (GenBank OK188782)
CaspaseI_Rq	TACCAGACGTACACCGCC			
Atg2_Fq	TGTGGCCCAGTGTGTCATTG	*B. trigonica* Atg2	147	This study (GenBank OK188779)
Atg2_Rq	CTGTTGCCTGTCTTGCCCTT			
Atg5_Fq	TGGCACTACCCTATTGGTCT	*B. trigonica* Atg5	205	This study (GenBank OK188780)
Atg5_Rq	TCTGCATGTTGGAAACAATCTG			
Atg16_Fq	AGAAGCAGCCAAGGACATGC	*B. trigonica* Atg16	157	This study (GenBank OK188781)
Atg16_Rq	CCTGTCCACAGGACTCCACT			
Primers used for detection (DNA)				
OA2-F (Lib16s_F)	GCG CTT ATT TTT AAT AGG AGC GGC A	*Ca.* L. solanacearum 16s rRNA	1168	111
OI2c-R (Lib16s_R)	GCC TCG CGA CTT CGC AAC CCA T			
Primers used for cloning				
ompA_full_F	ATGGGCAAAGAAAAAAAGGACTC	*Ca.* L. solanacearum OmpA (full length)	2331	This study
ompA_full_R	CTATCTTGCGGCATTCCCGA			
ompA_bait_F	CGCGAATTCATGGGCAAAGAAAAAAAGGACTC	*Ca.* L. solanacearum OmpA as bait (pGBKT7)		This study
ompA_bait_R	CGCGGATCCCTATCTTGCGGCATTCCCGA			
Omp-pFN2_sgf F	AGCTGCGATCGCCATGGGCAAAGAAAAAAAGGACT	*Ca.* L. solanacearum OmpA (Infusion cloning into pFN2A)		This study
Omp-pFN2_pme R	CGCGGTTTAAACCTATCTTGCGGCATTCCCGA			
Flagellin_F	ATGACTAGTATTCTAACTAATCTTCC	*Ca.* L. solanacearum Flagellin (full length)	1362	This study
Flagellin_R	CTAACCACGGAAAAGAGATAGAATTTTTG			
Flagellin_bait_F	CGCCATATGATGACTAGTATTCTAACTAATCTTCC	*Ca.* L. solanacearum flagellin as bait (pGBKT7)		This study
Flagellin_bait_R	CGCGGATCCCTAACCACGGAAAAGAGATAGAATTTTTG			
Flg_pFN2_sgf_F	GACCGCGATCGCCATGACTAGTATTCTAACTAATCTTCCC	*Ca.* L. solanacearum flagellin (Infusion cloning into pFN2A)		This study
Flg_pFN2_sgf_r	TTGTGTTTAAACCTAACCACGGAAAAGAGATAGA			
Vg_F	ATGTGGTCTCCAATCATCCTC	*B. trigonica* Vitellogenin (full length)		This study
Vg_R	TTAGGCGGCAACACATTTGG			
VWD_full_F	GAGAACCACAATTCCGTCAAT	*B. trigonica* Vitellogenin-VWD domain (full length)		This study
VWD_full_R	TTAGGCGGCAACACATTTGG			
VWD_pRSET_XhoI_F	CCCCTCGAGGAGAACCACAATTCCGTCAAT	Cloning VWD into pRSETA		This study
VWD_pRSET_EcoRI_R	CCCGAATTCTTAGGCGGCAACACATTTGG			
VWD_pGAD_F (EcoRI)	CCCGAATTCGAGAACCACAATTCCGTCAAT	Cloning VWD into pGADT7		This study
VWD_pGAD_R (XhoI)	CCCCTCGAGTTAGGCGGCAACACATTTGG			

### Recombinant protein expression, in vitro translation, and pulldown assay.

**(i) Prey (vitellogenin).** Full-length coding sequence for VWD domain of Vg was cloned into pRSET-A, and finally transformed into competent E. coli BL21(DE3) cells to express 6×His-Vg-VWD. The transformed clones were grown overnight in liquid LB at 37°C with agitation (200 rpm). A fresh media of 5 mL was seeded with 200 μL of this culture and grown for 4 h or until it reaches 0.6 optical density. At this point, a final concentration of 1 mM IPTG was added and was incubated with agitation at 30°C for additional 5 h. The cells were finally harvested at 6,000 rpm followed by protein extraction. The cells were resuspended in 200 μL of B-PER along with 1 μg of Lysozyme (Sigma-Aldrich) and DNase (Thermo), incubated for 15 min with shaking at room temperature and centrifuged at a high speed for 10 min. The supernatant was collected as purified protein and was used for further protein assays.

**(ii) In vitro translation of Bait protein.** Full-length CDS of OmpA/Flg was cloned into pFN2A (GST) Flexi vector separately for *in vitro* translation. Briefly, Liberibacter OmpA was amplified using SgfI and PmeI restriction sites (Table 1) and was digested using Flexi Enzyme Blend (Promega). Similarly, pFN2A was also digested and finally ligated to OmpA/Flg using T4 ligase (Thermo). The ligation mixture was used to transform E. coli DH5α for screening a positive recombinant pFN2A-OmpA/pFN2A-Flg vector. Following this, the recombinant plasmid was isolated and sequenced for confirmation. For *in vitro* translation, we used TNT Quick coupled transcription/Translation System (Promega) following the manufacturer’s instructions.

**(iii) Pulldown and Western blot assay.** The vector pFN2A was genetically modified to remove the Barnase gene and to express just the GST as control (Fig. S1). For pulldown assays, we used Magne-GST Pull-Down Systems (Promega, USA). The bait, pFN2A-OmpA/pFN2A-Flg, and control GST was immobilized onto Magne-GST particles following the manufacturer’s instructions. Total soluble fractions from prey protein lysate (6×His-Vg-VWD) was incubated with the bait immobilized to Magne-GST particles for capture. After washing, the bound proteins were finally eluted by boiling in 1 × SDS buffer, separated on 10% SDS-PAGE for analysis and detected by Western blot using monoclonal anti-polyHis antibody produced in mouse (Sigma-Aldrich, Israel).

**(iv) Homology modeling and in silico studies.** The open reading frames of the sequences derived from the yeast two-hybrid assays were annotated using BLASTp and NCBI Conserved Domain Database search (96) databases and checked for in-frame reading sequences. Structural analysis for domain identification for was done by Pfam (97) and NCBI-CDD. Full-length VWD domain of Vg was amplified from carrot psyllids and was used for all further studies. For phylogenetic studies, *Ca.* L. solanacearum full-length Vg sequence was aligned with 20 other insect Vgs in Mega 7.0 software with arachnid Vg as an outgroup. The phylogenetic relationship was assessed in CIPRES gateway using *M*_r_.Bayes XSEDE tool with fixed LG+G substitution model and 1 million generation. The tree was finally edited in Figtree program v1.4.4 (http://tree.bio.ed.ac.uk/software/figtree). Three-dimensional model structure for Vg was generated by iTasser (98) with the highest C-score. All known full-length sequences of OmpA/Flg from Liberibacter species were aligned in Mega7.0 (99) and similarity scores with consensus sequences were obtained in ESPript 3.0 (100). Three-dimensional model structure for OmpA was generated by Swiss-Model Tool (101) and the server Orientations of Proteins in Membranes (OPM) (https://opm.phar.umich.edu/) and for Flg by Swiss-Model.

### JH-III hormone treatment.

JH-III (Sigma, Israel), which is the principle regulator of Vg synthesis in Hemipterans (79, 102), was dissolved in ethanol at a concentration of 5 μg/μL. This concentration and application protocol was optimized after trying three different concentrations adapted from different reports. The one with low lethal activity and high effects on gene expression was chosen (103–107). To induce the expression of vitellogenin, JH-III was applied to a flush of parsley in an incubation box with 20 adult female psyllids (up to 1 week old with unknown mating status) for 16 h. Ethanol was used in the control set of experiment. The psyllids were collected and were used for oviposition, DNA/RNA isolation, and immunostaining analyses. The experiments were done in triplicate with minimum of six samples each for qRT-PCR/q-PCR.

### Induction and repression of autophagy.

Autophagy was induced by treating the psyllids with Rapamycin (Sigma, Israel) (a potent mTORC1 inhibitor). The experiment was set up similar to the dsRNA experiments as mentioned previously (94). Fresh leaf flush was placed in a microcentrifuge tube, applied with 10 μM Rapamycin (dissolved in ethanol). This concentration was again optimized based on low lethal effects. Twenty adult female psyllids (up to 1 week old) were released into each jar containing the leaf flush and were allowed to feed for 24 h. Similarly, Thapsigargin (Enco, Israel) was used for autophagy inhibition at a concentration of 10 μM and the application was similar to that of JH-III. Ethanol was used as control. DNA/RNA was extracted from the psyllids, midguts, and ovaries for qPCR and qRT-PCR analyses. Midguts were also used for immunostaining. Each experiment was conducted three times with a minimum of six samples each time for a total of minimum 18 samples.

### Immunolocalization.

Immunostaining for Vitellogenin, Liberibacter, and auto-lysosomes were done according to the protocol described previously (94). Psyllid midguts/ovaries were dissected out in PBS, fixed in 4% paraformaldehyde, treated with TritonX-100 and incubated in 1.5% blocking buffer for 1 h. Following this, the guts/ovaries were incubated with rabbit-polyclonal Anti-Vg antibody (Abcam) or Anti-OmpB antibody produced in rabbit (GenScript Corp., USA) (108) for 1.5 h followed by secondary antibody conjugated with Cy3/Cy5 counterstained with DAPI. The colocalization of Liberibacter and Vg was validated using the Colocalization Finder plugin of ImageJ with Pearson’s correlation coefficient (R value) using five different images (https://imagej.nih.gov/ij/plugins/colocalization-finder.html). LysoTracker Green DND-26 (Invitrogen) was used to locate auto-lysosomes according to the manufacturer’s instructions. At least eight midguts were used for each immunolocalization experiments to confirm the consistency of the results obtained. The differences in the signals for auto-lysosomes in *Ca.* L. solanacearum-free and *Ca.* L. solanacearum-infected midguts were validated using ImageJ software with area integrated intensity and mean gray value. A minimum of 10 images were used for measurement for each ImageJ analysis.

### Oviposition and egg hatching.

After JH-III treatment, the insects were released on fresh leaf flush for 72 h and the number of laid eggs (oviposition) by 10 female psyllids was counted on each leaf flush and were monitored for hatching (fertility). Eggs from *Ca.* L. solanacearum+ psyllids treated with ethanol were used as a control. To test the presence of transovarial transmission, the leaf flush (for control and JH-treatment) along with oviposited eggs were washed for 2 min in 0.05% bleach followed by 50% alcohol and finally washing in sterile distilled water thrice. The eggs were then carefully separated from the leaves from the pedicel with a sterile blade under a magnifying glass and placed on sterile *Ca.* L. solanacearum-uninfected leaves on a Petri dish (Fig. S2) and then incubated in the plant growth room. The newly hatched nymphs were allowed to feed on the uninfected leaves and were tested for *Ca.* L. solanacearum in the third instar stage. For control experiments, the eggs were placed on sterile *Ca.* L. solanacearum-infected leaves. The differences were analyzed by Student’s *t* test (*P* < 0.05).
