# Supplementary material for: Interaction of Liberibacter Solanacearum with Host Psyllid Vitellogenin and Its Association with Autophagy
Source: Microbiol Spectr. 2022 Jul 11;10(4):e01577-22. doi: 10.1128/spectrum.01577-22 (PMC9430699; doi:10.1128/spectrum.01577-22)
Supplement: Supplemental file 1 — Supplemental material. Download spectrum.01577-22-s0001.pdf, PDF file, 0.9 MB [file spectrum.01577-22-s0001.pdf]

Supplementary figures

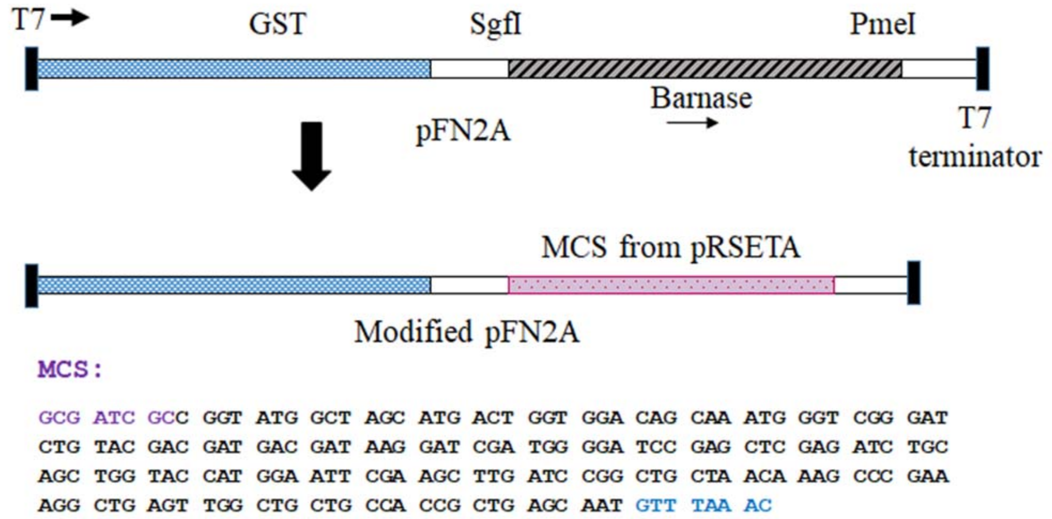

Fig. S1. Modified pFN2A vector showing the incorporated MCS removing the lethal Barnase gene.

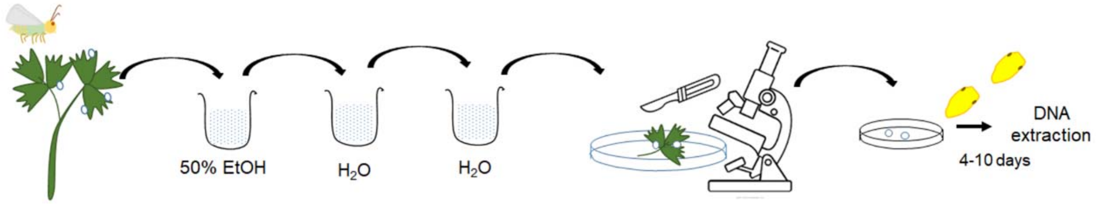

Fig. S2. Procedure by which eggs were separated from the leaves and were allowed to hatch in a sterile environment for CLso detection.

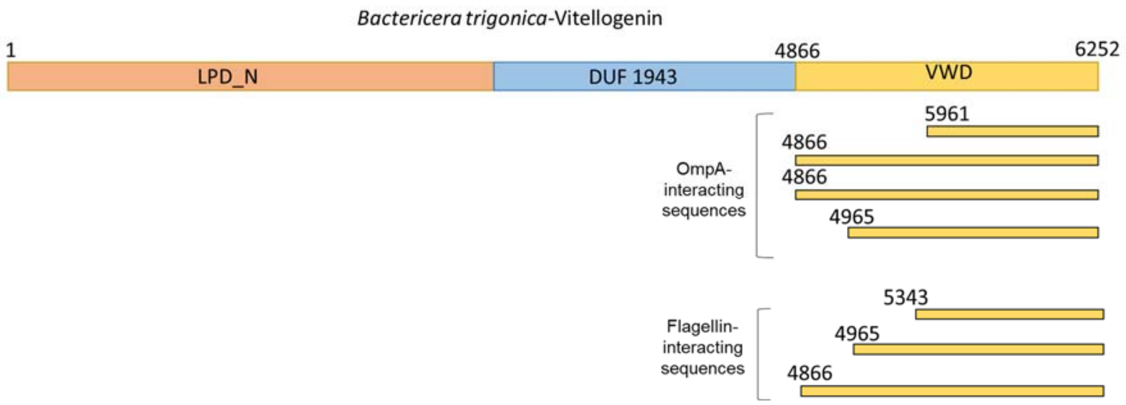

Fig. S3. Depiction of the sequences found in the Y2H assays when OmpA/Flg were used as baits.

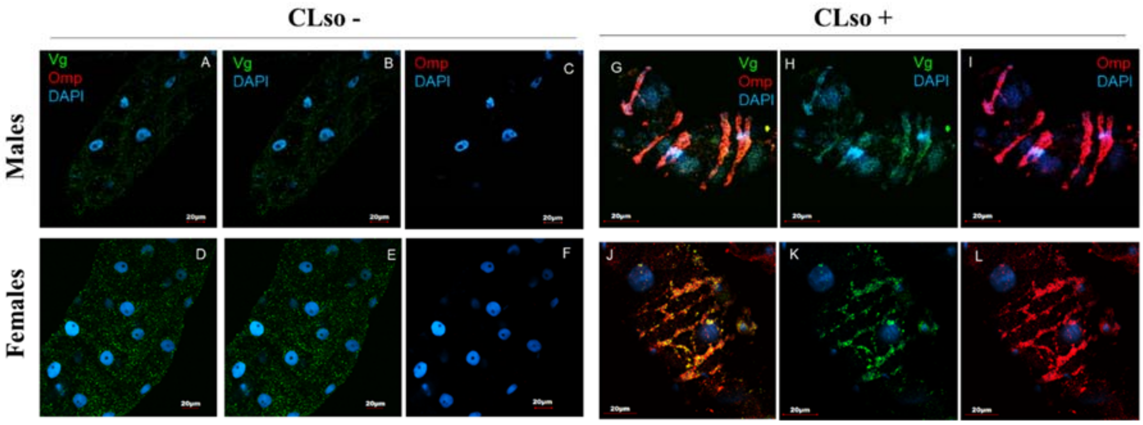

Fig. S4. Vitellogenin expression profile in Ca. L. solanacearum-free and Ca. L. solanacearum-infected male and female psyllid midguts using Immunolocalization. A-C, Ca. L. solanacearum-free male gut, D-F, Ca. L. solanacearum-free female gut, G-I, Ca. L. solanacearum-infected male gut, J-L, Ca. L. solanacearum-infected female gut. Red is CLso Omp, green is Vg and blue is DAPI staining of the nuclei.

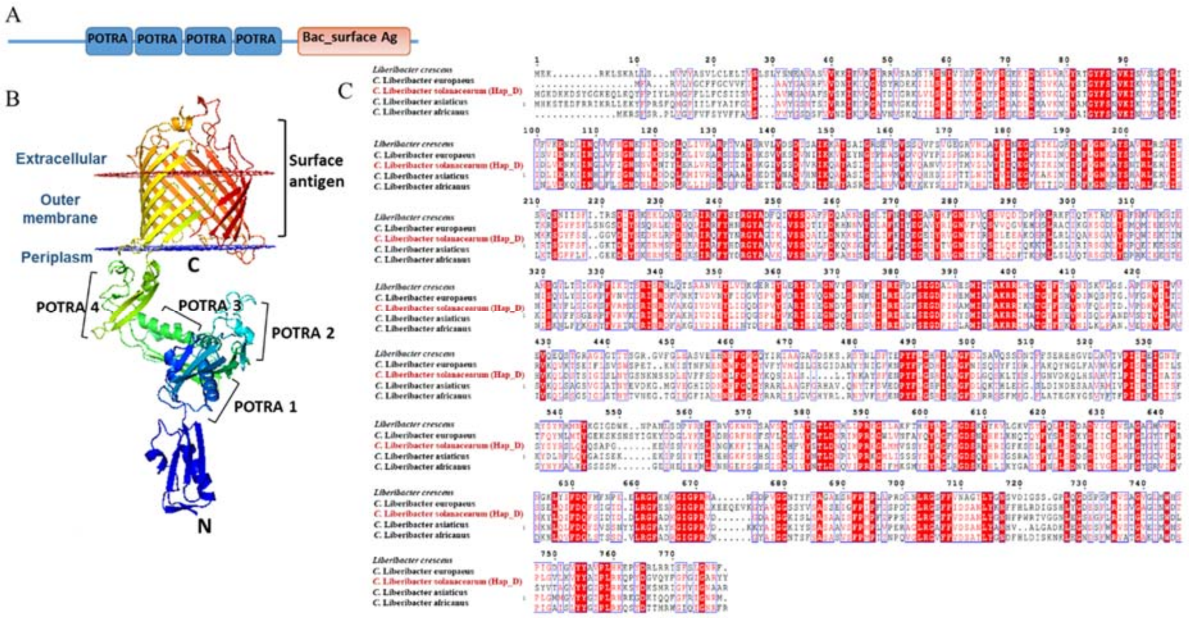

Fig. S5. Domain and conserved sequence analysis of OmpA. A and B, Presence of four polypeptide transport-associated domain (POTRA) and an extracellular surface antigen domain is conserved in Liberibacter OmpA. C, Multiple sequence alignment of known Liberibacter OmpA proteins showing conserved residues in red.

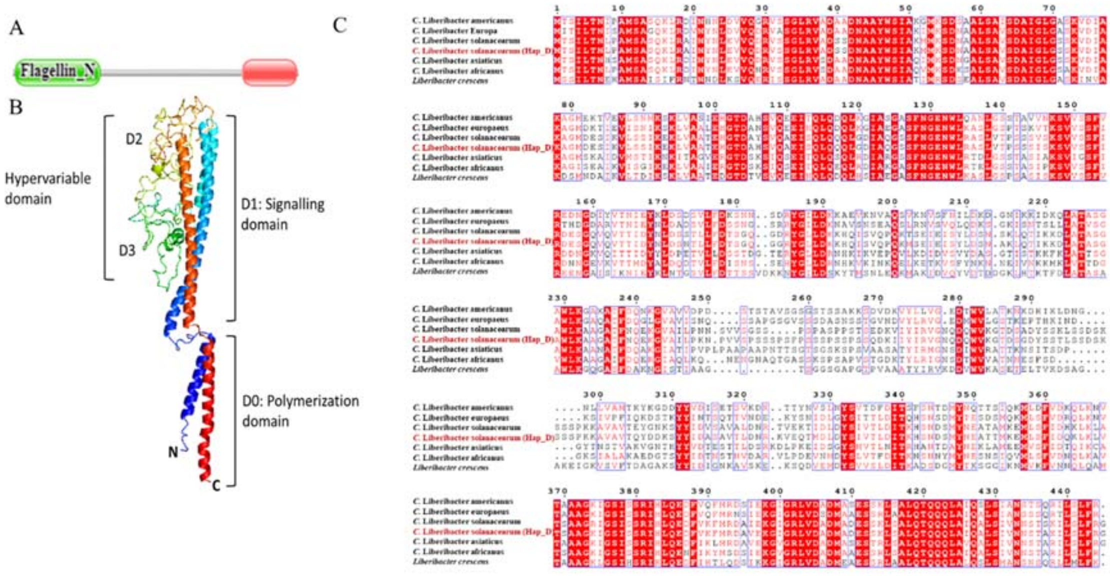

Fig. S6. Domain analysis of Flagellin. A and B, Flagellin structure showing signaling and polymerization domains. C, sequence alignment showing conserved residues across all known Liberibacter flagellins.

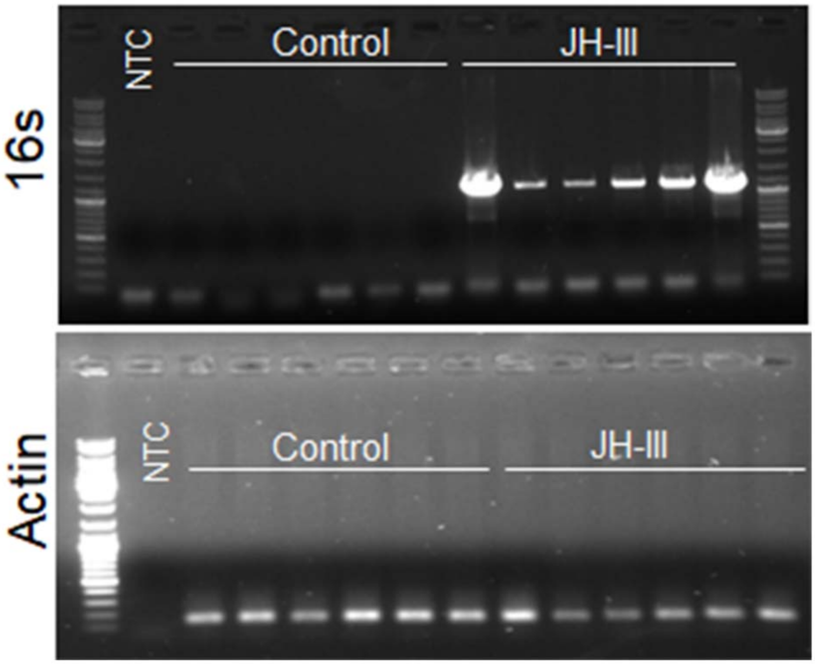

Fig. S7. PCR detection of CLso using 16s-rRNA specific primers and Actin as housekeeping control showing absence of CLso in L+ control ovaries and presence of CLso in JH-III treated ovaries.

Fig. 1D

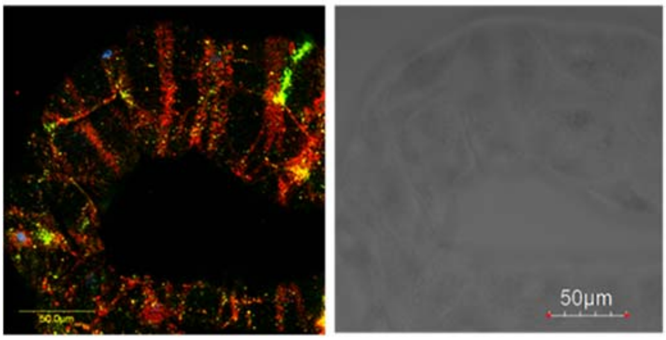

Fig. 3C

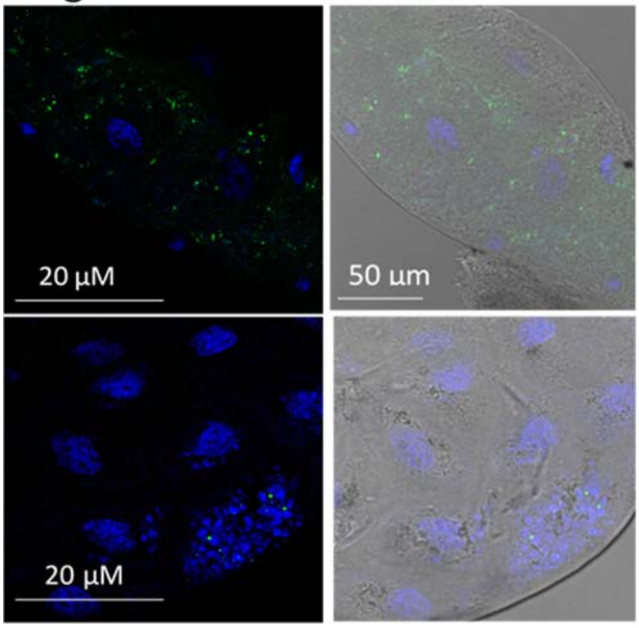

Fig. 5D

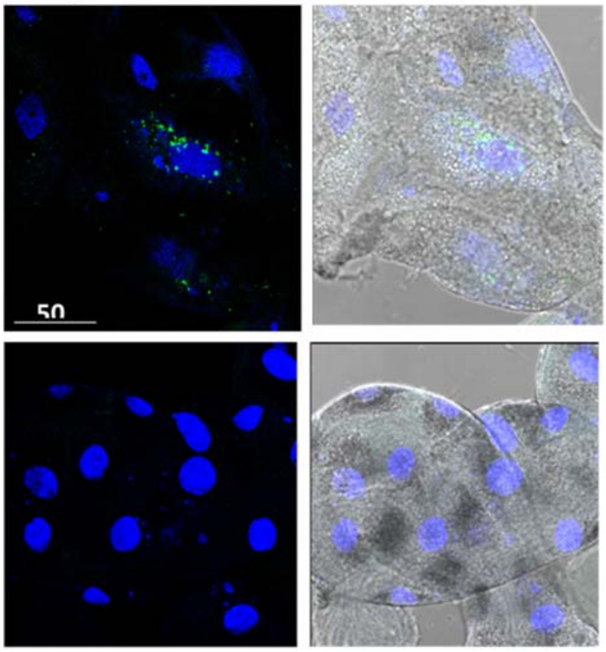

Fig. 3B

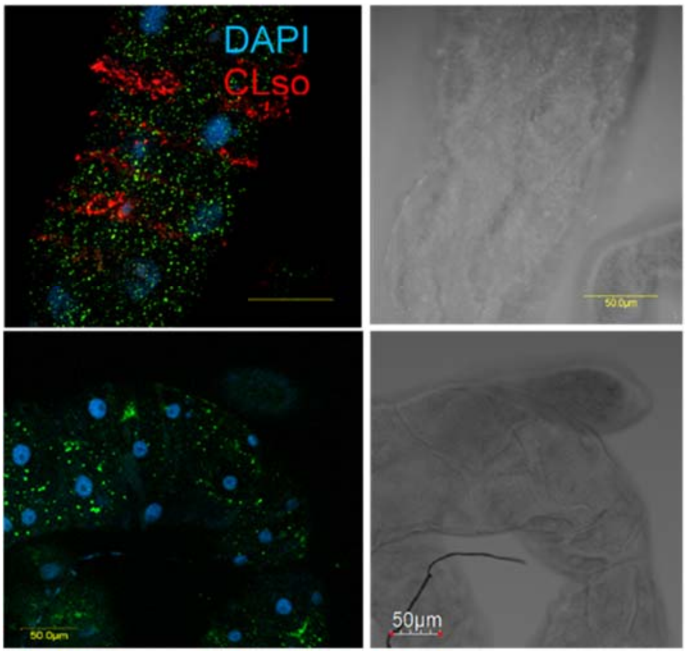

Fig. 4D

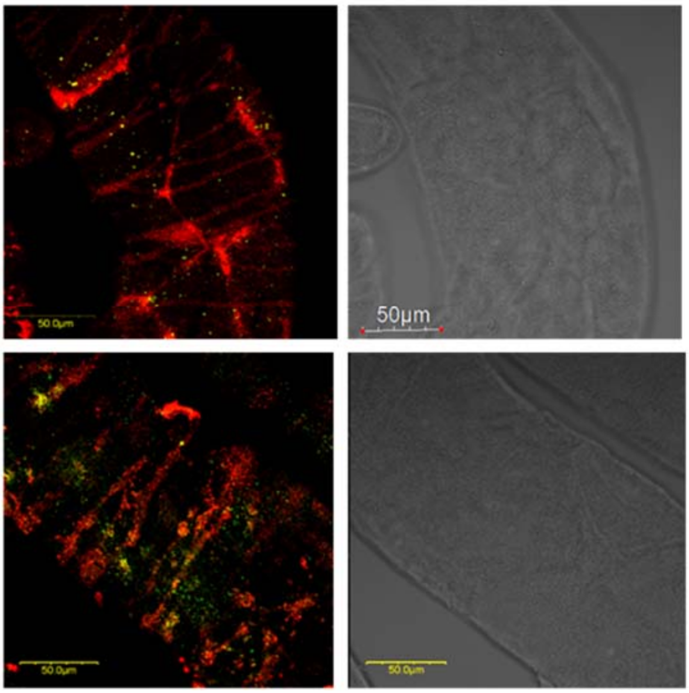

Fig. 6C

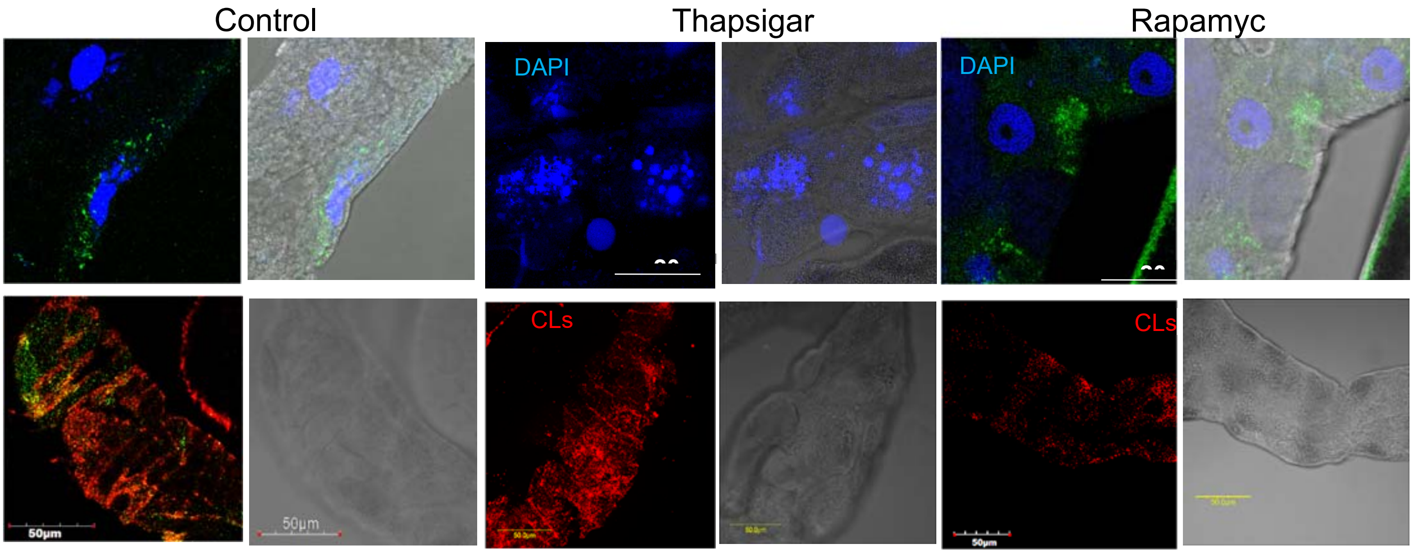

Fig. S8. Corresponding bright-field images for the all the confocal images of the psyllid midguts represented in Figures 1,3,4,5 and 6.
